# The American Surgical Association

**Published:** 1886-06

**Authors:** 


THE AMERICAN SURGICAL ASSOCIATION met in Washington, D.C., on May 28th and continued in session four days. Professor Moses Gunn presided. Many good papers were presented and discussed.

A proposition was considered by the Association to organize a CONGRESS, to be formed by the various SPECIAL SOCIETIES in the United States, and to meet annually in Washington. Its further discussion was deferred to the next annual meeting, and a committee was appointed to consider the proposition and to report next year.

By some it is charged that this is but an attempt to supplant the American Medical Association by those who no longer feel an interest in the prosperity of the old National Association, which it must be admitted represents more fully than any other in existence in our country, the whole medical profession in the United States.

